# Cross-Language Modulation of Visual Attention Span: An Arabic-French-Spanish Comparison in Skilled Adult Readers

**DOI:** 10.3389/fpsyg.2016.00307

**Published:** 2016-03-07

**Authors:** Faris H. R. Awadh, Thierry Phénix, Alexia Antzaka, Marie Lallier, Manuel Carreiras, Sylviane Valdois

**Affiliations:** ^1^Department of Psychology, Faculty of Arts, Al Qadisiya UniversityAl Diwaniyah, Iraq; ^2^LPNC, Laboratoire de Psychologie et NeuroCognition, Université de Grenoble-AlpesGrenoble, France; ^3^BCBL, Basque Center on Cognition, Brain and LanguageSan Sebastian, Spain; ^4^Centre National de la Recherche Scientifique, LPNC, UMR 5105Grenoble, France

**Keywords:** cross-language comparison, visual attention span, reading speed, reading direction, language transparency, letter string simultaneous processing

## Abstract

In delineating the amount of orthographic information that can be processed in parallel during a single fixation, the visual attention (VA) span acts as a key component of the reading system. Previous studies focused on the contribution of VA span to normal and pathological reading in monolingual and bilingual children from different European languages, without direct cross-language comparison. In the current paper, we explored modulations of VA span abilities in three languages –French, Spanish, and Arabic– that differ in transparency, reading direction and writing systems. The participants were skilled adult readers who were native speakers of French, Spanish or Arabic. They were administered tasks of global and partial letter report, single letter identification and text reading. Their VA span abilities were assessed using tasks that require the processing of briefly presented five consonant strings (e.g., R S H F T). All five consonants had to be reported in global report but a single cued letter in partial report. Results showed that VA span was reduced in Arabic readers as compared to French or Spanish readers who otherwise show a similar high performance in the two report tasks. The analysis of VA span response patterns in global report showed a left-right asymmetry in all three languages. A leftward letter advantage was found in French and Spanish but a rightward advantage in Arabic. The response patterns were symmetric in partial report, regardless of the language. Last, a significant relationship was found between VA span abilities and reading speed but only for French. The overall findings suggest that the size of VA span, the shape of VA span response patterns and the VA Span-reading relationship are modulated by language-specific features.

## Introduction

The visual attention (VA) span is defined as the number of distinct visual elements that can be processed simultaneously (at a glance) in a multi-element configuration (Bosse et al., 2007). VA span abilities are typically assessed through tasks of multi-character simultaneous processing (Lobier et al., 2012a). They are critical for reading (Ans et al., 1998). Participants with a larger VA span can allocate attention toward more letters simultaneously, thus identifying longer letter strings. Accordingly, VA span affects both word and pseudo-word reading. Larger VA span abilities relate to higher performance in irregular word reading (Bosse and Valdois, 2009), faster reading speed (Lobier et al., 2013) and weaker length effects (van den Boer et al., 2013), suggesting that a large VA span favors the fast whole-word procedure of reading. However, the VA span further relates to pseudo-word reading (Bosse and Valdois, 2009). Assuming that children with larger VA span can process more letters simultaneously, they should identify and process longer sublexical orthographic units as a whole (Valdois et al., 2004). Accordingly, Zoubrinetzky et al. (2014) showed that dyslexic children with abnormally reduced VA span tended to erroneously segment longer graphemes into shorter ones. VA Span abilities thus affect the size of relevant orthographic units that can be processed while reading.

The role of VA span in normal or atypical reading has been investigated in different languages –French (Bosse and Valdois, 2009; Lobier et al., 2012a; Zoubrinetzky et al., 2014), English (Bosse et al., 2007), Dutch (van den Boer et al., 2013), and Brasilian Portuguese (Germano et al., 2014). However, not only were previous investigations limited to European languages but VA span was typically assessed in each single language independently, thus preventing cross-language comparison. When VA span abilities were assessed in bilingual readers –French-Spanish (Lallier et al., 2013, 2014; Valdois et al., 2014a), French- Basque and Spanish-Basque readers (Lallier et al., 2016)–, findings suggested that learning to read in two languages that differ in transparency (e.g., French-Spanish or French-Basque; French is opaque but Spanish and Basque are transparent languages) affected VA span as compared to the performance of monolinguals or to that of bilinguals who were trained on languages with similar transparency (e.g., Spanish-Basque). These findings suggest potential modulation of VA span abilities depending on the language characteristics. In the current study, we will for the first time investigate VA span abilities in Arabic, a Semitic language, and directly compare VA span performance in Arabic and in two European languages, French and Spanish, that differ in transparency. Our purpose was to explore whether VA span abilities are modulated by the language characteristics and whether response patterns on VA span tasks are influenced by the direction of reading.

Cross-language studies on European languages mainly emphasized the distinction between transparent and opaque orthographies. Transparent orthographies are typically described as having simple and consistent one-to-one correspondences between graphemes and phonemes. Opaque orthographies have more complex correspondences so that the same spelling can be pronounced in many different ways (e.g., though, through, tough, cough). However, the transparent/opaque dichotomy further reflects differences in the size of relevant orthographic units. Graphemes are typically shorter in transparent languages while relying on larger units tends to make the orthography-to-phonology mapping more consistent in the opaque languages (Ziegler and Goswami, 2005). The French-Spanish comparison thus appeared as particularly relevant. In the shallow Spanish orthography, each grapheme typically represents a single phoneme and most Spanish graphemes are one letter long. In contrast in the deeper French orthography, many graphemes have more than one letter (e.g., “ou”, “eau”) and even one-letter graphemes are often context-dependent (like c, g, s), thus requiring more than one letter to be processed simultaneously.

Classification of the Arabic orthography on the transparent/opaque continuum is not straightforward (Saiegh-Haddad and Henkin-Roitfarb, 2014). The Arabic language can be written in two different orthographies. In vowelized Arabic, orthography-to-phonology relationships are fully transparent as diacritics are used to mark short vowels. The orthographic units are one character long (either letters or diacritics). This highly shallow orthography is used for beginning readers but the bulk of Arabic script is non-vowelized. Indeed in most Arabic texts, short vowels are missing so that the word orthographic sequence does not translate all phonemic information. It follows that the non-vowelized Arabic script can be viewed as a deep orthography, not because of inconsistent letter-sound relationships but because of the absence of orthographic markers for vowels. On the other hand in Arabic as in Semitic languages more generally, the morphological structure of words is critical for reading (Frost, 2012; Perea et al., 2014). Crucial morphological information about root morphemes (consonantal skeleton) and word patterns (mainly long vowels) is always fully represented in printed words, even in non-vowelized Arabic script. This regular and systematically represented morphological information increases the spelling-sound transparency of non-vowelized Arabic script. Root morphemes provide the core meaning of words whose phonological form is mainly inferred from the sentence or text context (Abu-Rabia, 1997).

We identified orthographic transparency as a first feature that might influence VA span abilities. Learning to read in a more opaque orthography might enhance the processing of larger sublexical orthographic units and modulate VA span abilities. Lallier et al. (2016) showed that VA span abilities differed in French-Basque and Spanish-Basque bilinguals. The French-Basque children showed more efficient abilities suggesting that the opaque French orthography boosted the use of larger units. Along this line of reasoning, Spanish and Arabic readers who have been trained to process smaller sub lexical units during reading acquisition might have a smaller VA span than French readers who had to process longer sub lexical units.

However, the size of VA span may also vary depending on the language orthographic processing constraints at the word level. It is well established that skilled readers of opaque languages process words as a whole. In English, Adelman et al. (2010) showed that the identification of all of the letters within a 4-letter word began simultaneously after only a few milliseconds of visual presentation. Similar findings were reported for 5-consonant strings in French (Marzouki and Grainger, 2014). No such direct evidence is available for skilled Spanish readers. However, studies on length effects (Suárez-Coalla and Cuetos, 2012), lexicality effects (Lallier et al., 2014) word frequency and neighborhood effects (Carreiras et al., 1997) in typical reading and evidence for differential processing of words and pseudo-words in dyslexia (Jiménez et al., 2009) suggest that words are processed as a whole in Spanish as in more opaque languages. Accordingly, VA span abilities should be similar in the French and Spanish languages that both require the whole word letters to be processed in parallel but might differ in the Arabic language. Indeed, word recognition in Semitic languages is primarily based on the processing of (3-consonant long) root morphemes (Farid and Grainger, 1996; Deutsch and Rayner, 1999; Perea et al., 2010; Velan and Frost, 2011; Boudelaa, 2014). Skilled readers thus develop expertise for the identification of root letters but do not demonstrate whole word parallel processing skills (Abdulhadi et al., 2011; Boudelaa, 2014).

The use of different alphabets in French/Spanish and Arabic could further differently affect performance in the letter report tasks we used to assess VA span. Previous research on single letter processing has shown that Arabic letters are more difficult to identify than Latin letters (Ibrahim et al., 2002, 2007; Abu-Rabia and Taha, 2004; Pelli et al., 2006). Even skilled Arabic readers show slower reaction times for processing Arabic than Latin letters (Carreiras et al., 2013; for a review: Eviatar and Ibrahim, 2014). Letter string processing in the Arabic participants may therefore partly reflect the lower discriminability of Arabic than Latin letters. As a consequence, VA span abilities might be reduced in Arabic as compared to Spanish or French. Moreover, VA span performance should relate to single letter identification skills in all three languages. To summarize, we identified three potential sources of between-language variation in VA span due to differences in the size of sublexical units, in whole-word vs. root-based orthographic processing or in letter discriminability. A second issue was whether VA span response patterns are affected by the direction of reading.

Several studies have explored the influence of reading direction on the perceptual span, defined as the area of text from which useful information is extracted during a fixation (Rayner, 1986). The perceptual span is typically measured using the gaze-contingent moving window paradigm. In this paradigm, a region of text delineated by a window extending around gaze position is normally displayed but the text outside the window is obscured. It has been shown that reading rate is normal in English when the moving window extends asymmetrically to the right. Actually, an asymmetry of the perceptual span to the right is reported in left-to-right languages (Rayner, 1986, 2009) but a reversal in the asymmetry is found for right-to-left languages, like Hebrew (Pollatsek et al., 1981) or Urdu (Paterson et al., 2014). Accordingly, English-Arabic bilinguals show superior reading performance in Arabic for windows extending to the left but an advantage for an asymmetry to the right when reading English (Jordan et al., 2014). However, such effects might primarily reflect eye movement processing constraints. Perceptual span asymmetry in the direction of reading enhances parafoveal processing that provides information on upcoming words and contributes to the programming of the subsequent saccadic eye movement. Thus, studies on the perceptual span do not provide strong evidence for reading direction effects at the word level.

Other sources of evidence suggest that reading habits may further modify the way we process individual words and consonant strings. It is well documented that printed word recognition performance in skilled readers varies as a function of fixation location within the word. This effect known as the optimal viewing position effect (Nazir et al., 1992; O'Regan and Jacobs, 1992) is characterized by a left-right asymmetry in the European languages. The probability to accurately recognize a word in a single fixation is highest when the initial fixation is left rather than right of the center of the word. The asymmetry is less clear for languages read from right to left (Brysbaert and Nazir, 2005). The optimal viewing position effect was reported as more symmetric in Arabic, due to divergent left-right asymmetries depending on the morphological structure of the stimuli (Farid and Grainger, 1996). However, opposite left-right asymmetry has been reported depending on reading direction in a task of single letter identification within string (Nazir et al., 2004). Latin letters were better identified when displayed leftward of the center of the string but a rightward advantage was reported for the Hebrew letters. These overall findings would suggest an influence of reading direction on the processing of non-lexical letter strings.

In the current paper, we will use the letter-string simultaneous processing tasks of global and partial letter report to assess VA span abilities (Bosse et al., 2007; Bosse and Valdois, 2009; Prado et al., 2007; Lassus-Sangosse et al., 2008; Lobier et al., 2012a; Valdois et al., 2012). A sequence of 5 consonants is briefly displayed centered on fixation and participants are asked to report all of the string letters in the global condition or a single letter whose location is indicated by a retro-cue displayed at the offset of the consonant string in the partial condition. An effect of reading direction on the VA span response profiles would predict a leftward advantage on letter identification in French and Spanish but a rightward advantage in Arabic. Based on previous evidence of the robustness of the influence of reading habits—that extends to various tasks using letters or words and to non-linguistic contexts, as perceptual judgment (Morikawa and McBeath, 1992), aesthetic judgment (Chokron and De Agostini, 2000), perception of facial affects (Vaid and Singh, 1989) or spatial representation of numbers (Shaki et al., 2009)—an effect of reading direction should generalize to the two global and partial conditions of the VA span tasks.

To summarize, this study follows two main goals. We will first explore whether VA span abilities vary in French, Spanish and Arabic adult readers depending on language differences in transparency, orthographic processing or letter complexity. Second, we will investigate whether VA span performance pattern in global and partial report is similarly affected by the direction of reading. The participants were further administered a text reading task to explore the relationship between VA span and text reading performance in all three languages.

## Materials and methods

### Participants

The 123 participants were skilled adult readers of the Arabic, French or Spanish languages. They all were native speakers of one of these three languages. All had some basic knowledge of another language (English for the French participants; French for the Arabic participants; Basque (*N* = 18) or English (*N* = 24) for the Spanish participants) that they learned as a second language, during secondary school or at the adult age for the Arabic-speaking Iraqi participant. All participants were University students. The French and Spanish participants were undergraduate students recruited in France and Spain respectively. The Iraqi participants were high-level Ph.D students who were supported by grants from the Iraqi government. They were residents in France for their University education at the time of testing and had benefited from only 1-year formal education on the French Language. Their knowledge of French and their French reading skills were thus minimal. None of the participants reported any learning difficulty during childhood. All were right-handed and had normal or corrected-to-normal vision. The Arabic group of 42 participants (19 females) had a mean age of 29.4 years (*SD* = 6.7). The mean age of the French group of 39 participants (24 females) was 22.5 (6.1). The Spanish participants (*N* = 42, 32 females) were 22.3 (2.3) years old on average. The two French and Spanish groups did not differ in chronological age (*F* < 1). The Arabic group was slightly older than the other two groups [*F*_(1, 120)_ = 46.7, MSE = 28.9, *p* < 0.0001]. All the participants were tested in their mother tongue. Written informed consents were obtained from all participants. The study was approved by the local Ethics Committee of the Université Grenoble-Alpes.

### Experimental tasks

#### The VA span tasks

##### Stimuli

Random five letter-strings were built up from 10 consonants (“B, P, T, F, L, M, D, S, R, H”) of the Latin alphabet for French and Spanish; 10 consonants^1^ were also used in Arabic:


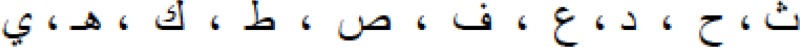


The letters were presented in black on a white background. Upper case letters (Arial, 7 millimeters high) were used in French and Spanish. A single set of letters is available in Arabic (no upper/lower case distinction). The Arabic letters were formatted to have the same size as the Latin letters. The spaces between adjacent Latin and Arabic letters was increased of 0.57° to minimize crowding effects. The 5-letter strings were of similar length in the three languages (e.g., R H S D M; angular size = 5.4°). They contained no repeated letters and never matched the skeleton of a real word in either French or Spanish (e.g., FLMBR for FLAMBER “burn”). Two adjacent letters never corresponded to an existing grapheme (e.g., PH, TH) or a frequent bigram (e.g., TR, PL, BR). The Arabic consonant strings never included an existing root morpheme in Arabic. Twenty 5-letter strings were displayed in Global Report. Each letter was used 10 times and appeared twice in each position. Fifty random 5-letter strings were used in Partial Report. Each letter occurred 25 times (5 times in each position).

##### Procedure

**Figure 1:**
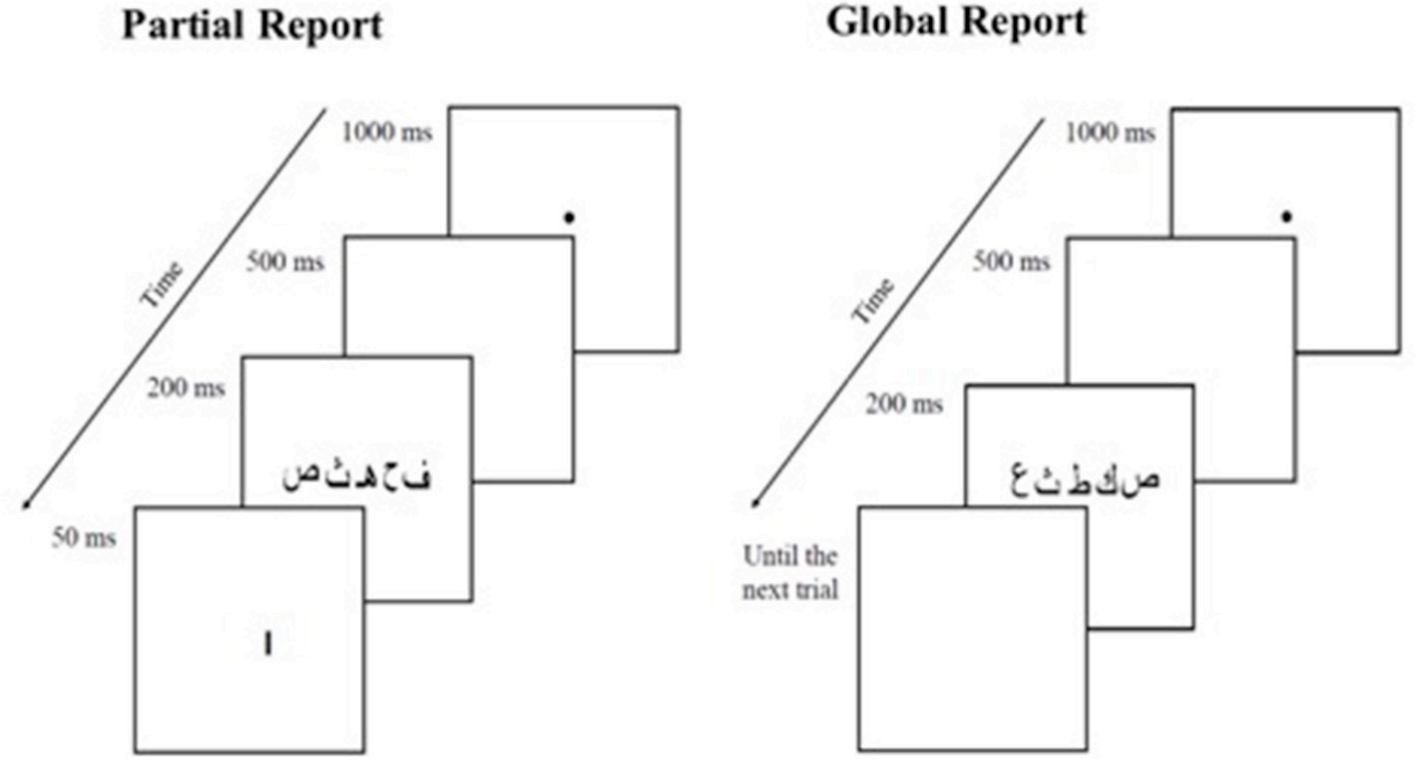
**Schematic illustration of the global and partial report tasks for the Arabic language**.

At the beginning of each trial, a central fixation point was presented for 1000 ms followed by a blank screen for 50 ms. Then, a 5-consonant string centered on fixation was displayed for 200 ms, a duration that corresponds to the mean duration of fixations in reading (Rayner, 1986). A 200 ms display is long enough for an extended glimpse, but too short for additional extraction of orthographic information following a saccadic eye movement. In the global report condition, participants had to report verbally as many letters as possible immediately after the string disappeared. In partial report, a vertical bar (i.e., a retro-cue) indicating the letter to be reported was displayed 1.1° below the target letter, at the offset of the letter-string. Each letter was used as target once in each position. Participants were asked to report the cued letter only. In both tasks, the experimenter pressed a button to start the next trial after the participant's oral response. The experimental trials were preceded by 10 training trials for which participants received feedback. No feedback was given during the experimental trials. The dependent measure was the percentage of letters accurately reported (identity not location) across the 20 trials in global report or across the 50 trials in Partial report. A description of the task is provided on Figure 1 for the Arabic language.

#### Single letter identification threshold

To assess single letter identification in the three languages, each of the 10 consonants used in the report tasks were randomly presented (5 times each) with the same physical characteristics as in the experimental tasks, at 5 different presentation durations (33, 50, 67, 84, and 101 ms). At the offset of the letter, a mask (13 mm high, 37 mm wide) was displayed for 150 ms. Participants were asked to name each letter immediately after its presentation. The test trials were preceded by 10 practice trials (2 for each presentation time) for which participants received feedback. The identification threshold corresponded to the shorter duration for which at least 80% letters were accurately identified.

#### Text reading

The texts in French and Spanish were taken from newspapers (from “Le Monde” and “El Mundo” respectively). The French text is descriptive and reports an artistic experience in Moscow during the twentieth century. The text is part of the ETULEC battery for the reading level assessment of French University students (Lassus-Sangosse, 2012). The Spanish text was chosen in order to match the French text in length and difficulty (include proper names, similar syntactic level); its topic was climate change. The Arabic text reports the biography of an Iraqi archeologist (Taha Baqir). The text was built up for the need of the experiment. Iraqi linguists judged the Arabic text appropriate for skilled adult readers. Most words were presented without diacritics except for a few words that required diacritics to be disambiguated. For all three languages, the text was presented in black on a white sheet (24 × 21 cm). The three texts are provided as Supplementary Material. The participants were asked to read the text aloud for a maximum of 3 min. The number of words accurately read per minute was computed for each participant.

The participants were tested individually in a quiet dimly lit room in a single session. The VA span tasks, reading task and single letter processing task were presented in a random order that varied from one participant to the other in each language group.

## Results

The overall descriptive data on the VA span tasks of global and partial report, single letter identification threshold and text reading is presented for the three groups of participants in Table 1.

**Table 1:** **Performance (mean and SD) of the Arabic, French, and Spanish participants in global and partial report, text reading and single letter identification**.

**Participants**	**Global report (max** = **100)**	**Partial report (max** = **50)**	**Text reading (wpm)**	**Letter identification threshold (ms)**
Arabic	72.17 (10.14)	37.52 (7.92)	110.67 (17.50)	54.07 (16.26)
French	92.82 (6.63)	45.77 (3.84)	157.58 (16.64)	33.31 (2.31)*^#^*
Spanish	93.62 (6.08)	46.09 (3.91)	167.43 (25.31)	33.81 (3.66)

# *Taken from the EVADYS norms (Valdois et al., 2014b)*.

### Cross-language VA span comparison

As shown on Table 1, French and Spanish participants reported more letters than Arabic participants in global and partial report. In global report, 92.8 and 93.6% Latin letters were reported on average for the French and Spanish participants respectively, against only 72.2% for the Arabic participants. Performance in partial report was very similar with an average of 91.5 and 92.2% accurate report in French and Spanish as compared to 71% in Arabic. ANOVAs with Language as the independent variable were performed separately for each task. The effect of Language was significant for the two VA span tasks of global report [*F*_(2, 120)_ = 99.65, MSE = 61.66, *p* < 0.0001, η^2^ = 0.62] and partial report [*F*_(2, 120)_ = 31.26, MSE = 31.36, *p* < 0.0001, η^2^ = 0.34]. Planned comparisons showed that performance of the French and Spanish participants was similar in global and partial report [for the two tasks, *F*_(1, 120)_ < 1] but differed significantly from that of the Arabic participants (Global report: [*F*_(1, 120)_ = 199.09, MSE = 61.66, *p* < 0.000001, η^2^ = 0.62]; Partial report: [*F*_(1, 120)_ = 62.45, MSE = 31.36, *p* < 0.0001, η^2^ = 0.34]). Thus, the size of the VA span is similar in French and Spanish but Arabic readers have a smaller VA span.

The participants' mean single letter identification threshold is reported on Table 1 for the Arabic and Spanish languages. The French data were lost due to technical problems and the French norms taken from young adults of the same age in the EVADYS test (Valdois et al., 2014b) are provided in Table 1, suggesting very similar abilities in French and Spanish. Comparison of Spanish and Arabic single letter processing skills showed that isolated letters were identified faster by the Spanish than the Arabic participants [*F*_(1, 82)_ = 62.07, MSE = 138.89, *p* < 0.0001, η^2^ = 0.43]. To assess whether VA span performance was related to single letter processing skills in the Arabic group, a composite score of single letter processing was computed as the weighted sum of letter identification performance at each of the five display durations (score at 33 ms ^*^ 5 + score at 50 ms ^*^ 4 + score at 67 ms ^*^ 3 + score at 84 ms ^*^ 2 + score at 101 ms). No significant correlation was found between this score and performance obtained either on the global (*r* = −0.04, *p* = 0.78) or on the partial (*r* = 0.03, *p* = 0.86) report tasks.

### Left-right asymmetry of the VA span in the three languages

**Figure 2:**
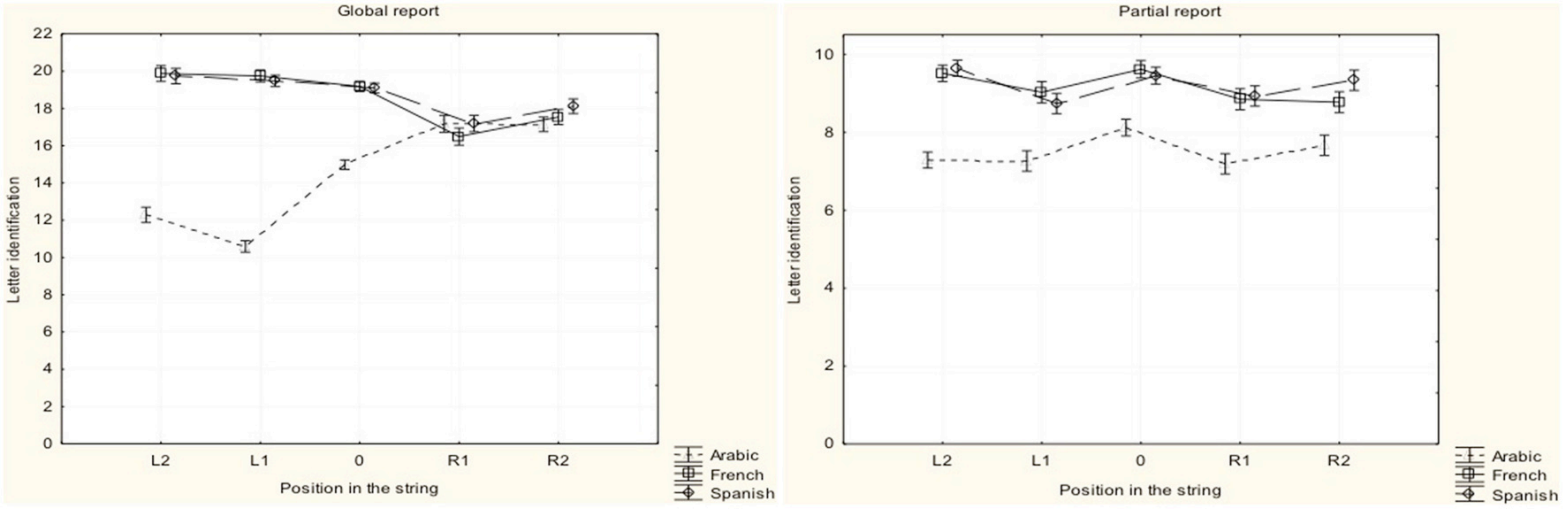
**Response patterns on the VA span tasks of global and partial report for the Arabic, French and Spanish participants**. Letter position labels are provided by reference to the fixation point, regardless of reading direction. 0, fixation point; L2, L1, leftward positions; R1, R2, rightward positions.

The response patterns of the three language groups in global and partial report are presented in Figure 2.

Figure 2 shows that performance in global report is characterized by a left-to-right gradient in French and Spanish but a right-to-left gradient in Arabic. Performance does not seem sensitive to the left or right side position of the target in the partial report condition. To more directly assess left-right asymmetry in global and partial report, two composite scores were computed as the summation of the identification scores for the two leftward (L1+L2) and the two rightward (R1+R2) letters of the 5-letter strings. ANOVAs were computed on these values with Language as the between-subject factor and Laterality as the within-subject factor. Results from Global Report (Figure 2) showed a significant Language x Laterality interaction [*F*_(2, 120)_ = 112.45, MSE = 32.31, *p* < 0.00001 η^2^ = 0.65], a main effect of Language [*F*_(2, 120)_ = 82.45, MSE = 12.01, *p* < 0.00001, η^2^ = 0.58] but no main effect of Laterality [*F*_(1, 120)_ = 1.58, MSE = 32.31, *p* = 0.21, η^2^ = 0.005]. Planned comparisons revealed a similar Laterality effect in French and Spanish [*F*_(1, 120)_ = 1.78, MSE = 32.31, *p* = 0.19, η^2^ = 0.005] but a highly significant difference between these two groups and the Arabic group [*F*_(1, 120)_ = 223.12, MSE = 32.31, *p* < 0.000001, η^2^ = 0.65]. A left-right asymmetry characterized performance for the French [*t*_(38)_ = 5.69, *p* < 0.00001] and Spanish [*t*_(41)_ = 5.79, *p* < 0.00001] groups whereas performance of the Arabic readers was characterized by a right-left asymmetry [*t*_(41)_ = −11.62, *p* < 0.00001].

Results from the Partial Report task are presented in Figure 2. Contrary to Global Report, there was no significant Language by Laterality interaction [*F*_(1, 122)_ = 2.51, MSE = 6.27, *p* = 0.11, η^2^ = 0.04]. The main effect of Language was significant [*F*_(2, 121)_ = 30.92, MSE = 5.50, *p* < 0.00001, η^2^ = 0.34] but the effect of Laterality was not [*F*_(1, 122)_ = 1.17, MSE = 6.27, *p* = 0.28, η^2^ = 0.02].

### Relationship between VA span and reading performance

As shown on Table 1, text reading performance showed that more words were processed per minute in French and Spanish than in the Arabic language. An ANOVA with Language as the independent variable showed a main effect of Language on reading speed [*F*_(2, 120)_ = 93.33, MSE = 3702.12, *p* < 0.0001, η^2^ = 0.61]. This effect was mainly due to the fact that Arabic participants were slower in text reading than the French [*F*_(1, 79)_ = 152.22, MSE = 2630.59, *p* < 0.0001, η^2^ = 0.66] or Spanish participants [*F*_(1, 82)_ = 142.83, MSE = 4262.31, *p* < 0.0001, η^2^ = 0.64]. Reading speed was slightly higher in Spanish than in French [*F*_(1, 79)_ = 4.21, SME = 4192.2, *p* = 0.044, η^2^ = 0.05].

**Figure 3:**
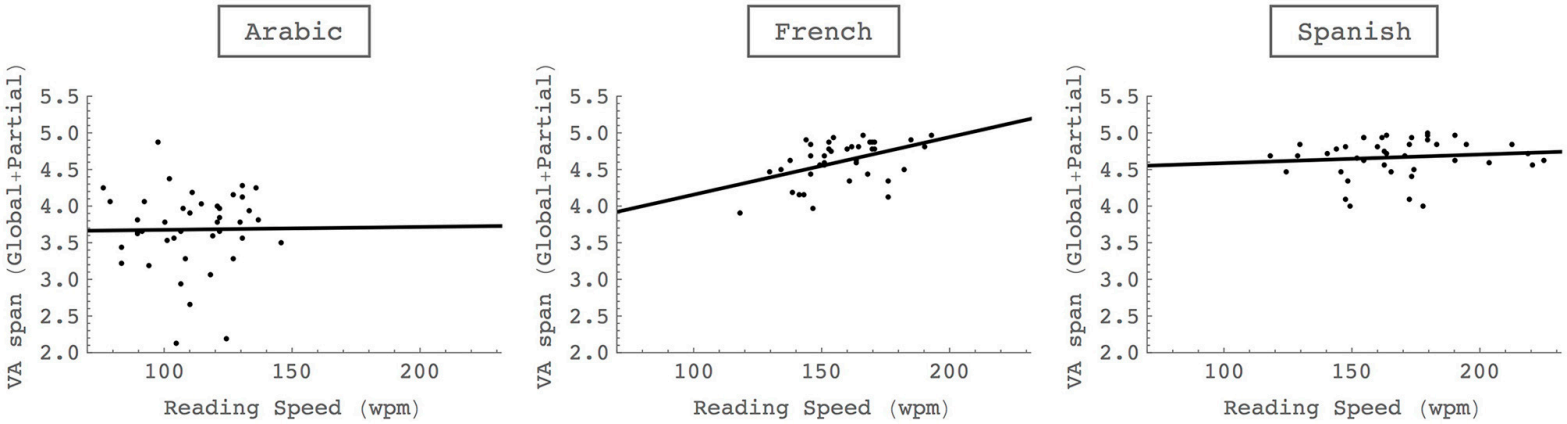
**Correlations between VA span and text reading speed in French, Spanish and Arabic**.

Correlation analyses were computed between text reading performance and VA span skills for the three languages separately. A composite score of VA span was computed from performance in global and partial report reduced to the mean number of letters accurately processed at each trial. The scatterplots are provided on Figure 3 for the three languages and the two conditions of letter report.

As shown on Figure 3, the correlation between VA span performance and reading speed was only significant for French (*r* = 0.46, *p* = 0.003) but not for Spanish (*r* = −0.11, *p* = 0.49) and Arabic (*r* = −0.013, *p* = 0.94). Otherwise, no correlation was found between text reading speed and single letter identification skills neither for Arabic (*r* = 0.063, *p* = 0.69 at 33 ms) nor for Spanish (*r* = 0.162, *p* = 0.31 at 33 ms).

## Discussion

It can be assumed that the same cognitive mechanisms are involved in reading in all languages whatever their transparency or writing system (Frost, 2012). However, the cognitive mechanisms of reading must be tuned to language-specific characteristics. Here, we focused on the VA span, i.e., a cognitive process of the reading system involved in letter-string processing (Bosse et al., 2007; Bosse and Valdois, 2009; Lobier et al., 2013). For the first time, we assessed VA span abilities in Arabic readers and compared their performance with that of French and Spanish readers to directly explore the influence of language characteristics on VA span. The main results can be summarized as follows. First, French and Spanish adult readers showed similar VA span abilities. Despite their higher University degree, the Arabic participants showed reduced VA span as compared to the two other language groups. Second, VA span patterns were characterized by a left-right asymmetry in the condition of global report. Leftward letters were better identified than rightward letters in French and Spanish but a rightward letter advantage was found in Arabic. No left-right asymmetry characterized performance in partial report in any language. Last, French and Spanish young adults read texts faster than the Arabic readers but only the French participants showed significant correlation between VA span and text reading speed.

### Variations in the size of VA span

A first key finding of our study is that VA span abilities are poorer in Arabic readers as compared to French and Spanish readers who otherwise performed similarly on the VA span tasks. These findings clearly rule out any potential impact of language transparency on VA span. Although French and Spanish readers have learnt to process sublexical orthographic units of different length during reading acquisition, their VA span is not differently sized at the adult age. Note that previous report of VA span size modulation depending on the second language transparency in balanced bilingual children suggests that transparency might be a relevant factor at an earlier age (Lallier et al., 2016).

Lower VA span in Arabic readers may reflect differences in letter complexity. Many behavioral studies have shown that the identification of Arabic letters is more difficult than the identification of Latin letters (Eviatar et al., 2004, for a review) and may be more attention demanding (Abu-Rabia, 2001; Hansen, 2014). If the amount of VA required for individual letter processing is greater in Arabic, then fewer letters should be simultaneously identified in this language than in French or Spanish. In line with this reasoning, we did found faster single letter identification in Spanish than Arabic readers. However, if single letter processing efficiency is the main source of variation in VA span, then we would expect significant correlations between single letter identification and VA span skills. To the contrary, no significant relationship was found between these two skills in the Arabic group, suggesting that their lower single letter processing skills were not the primary source of their poor VA span abilities. Further research should address this issue more deeply. Previous studies have shown that VA span abilities may be similarly measured using arrays of letters or digits in oral report tasks (Valdois et al., 2012) or in string comparison tasks (Reilhac et al., 2013) and that similar estimations are obtained using non-alphanumeric material in non-verbal categorization tasks (Lobier et al., 2012a). Cross-language VA span comparison on tasks using Arabic digits or non-alphanumeric multi-character strings would provide useful insights on whether Arabic readers intrinsically show reduced VA span as compared to the readers of European languages.

VA span differences in French/Spanish vs. Arabic could also reflect cross-language word orthographic processing differences. In Arabic as in all Semitic languages, word identification mainly relies on the processing of root morphemes that convey the core meaning of words (Frost, 2012; Boudelaa, 2014; Perea et al., 2014). It follows that all the Arabic word constituent letters do not have the same impact on word identification. Most relevant information comes from the three (or four) letters of the root morpheme. So, Arabic word recognition may oblige the reader to rely on information below the word level and focus on the few letters that carry information on root identity and meaning (Perea et al., 2010). In line with this hypothesis, some findings suggest that even skilled readers do not use a global word-form strategy when processing Arabic words (Boudelaa, 2014). This contrasts with the European languages for which global processing is the hallmark of reading proficiency. In these languages, all letters contribute to word identity, thus requiring attention to spread over the whole word letter-string. If we assume that VA span is tuned to the processing of relevant orthographic information for word identification, then French and Spanish readers who have to process the whole letter-string simultaneously may show larger VA span abilities as compared to the Arabic readers tuned to process root information first while reading. It is nevertheless clear that more empirical work is required to support this account. In particular despite lower complexity of the Hebrew than Arabic letters, such an account would predict similarly low VA span abilities in Hebrew and Arabic readers, which remains to be demonstrated.

### The effect of reading direction

The second main finding of the current research is that performance is characterized by a left-right asymmetry of opposite directionality in Arabic as compared to French and Spanish for the global report condition but no left-right asymmetry for any language in the partial report condition. Evidence for an asymmetry in the direction of reading in global report is consistent with previous research showing that experience with reading modifies the way we process information, in general (Chokron and De Agostini, 2000; Shaki et al., 2009) and letters within strings in particular (Nazir et al., 2004). However, the well-documented robustness of reading direction effects would have predicted similar left-right asymmetries in partial report, which was not found. Evidence for a left-right asymmetry in global report but no asymmetry in partial report suggests that a simple effect of reading experience cannot serve as explanation for the current findings. Such task-dependent effects cannot be accounted for either by the explanations previously proposed to account for left-right asymmetries, namely effects of lexical constraints (the fact that word beginnings are more informative) or hemispheric specialization (the fact that the leftward and rightward letters do not project in the same hemisphere). What underlying mechanism(s) may account for the task-dependent effects observed here?

Two previous findings may be informative to tentatively answer this question. First, very similar VA span estimations are typically obtained through global and partial report tasks with strong correlations between the two measures (Bosse et al., 2007; Bosse and Valdois, 2009; Valdois et al., 2014b)^2^, which suggests that the same mechanisms are at play in the two tasks. Previous studies in particular showed that interpretations in terms of poor verbal short-term memory (Lassus-Sangosse et al., 2008) or phonological recoding (Valdois et al., 2012) are unlikely to specifically account for performance in global report. Second, previous research on VA span showed (1) that VA span performance mainly reflects the VA capacity available for processing (Lobier et al., 2013) and (2) that the cerebral correlates of VA span are attentional brain regions located in the superior parietal lobule bilaterally (Peyrin et al., 2011, 2012; Lobier et al., 2012b; Reilhac et al., 2013). Accordingly, VA span appears a good candidate to account for the current findings. When the task requires all the letters to be accurately identified as in global report, VA deploys over the whole letter string to trigger each letter parallel processing. Even in case of parallel processing, covert attention bias acquired through reading practice may favor the processing of leftward letters in left-to-right languages but rightward letters in those languages with the opposite reading direction. The current interpretation is quite compatible with Eviatar (1995)'s claims that covert attention influences the processing of letter arrays when presented bilaterally with differential effects depending on reading habits.

Deployment of attention over the whole letter string is also required in partial report, but attention orienting is manipulated by providing a spatial cue at the offset of the 5-letter string. The retro-cue indicates the location of the single letter to be reported while the physical stimulus is absent so that the target letter has to be retrieved from memory. Recent studies have shown that retro-cues modulate the allocation of attention in working memory (Kuo et al., 2012; see Gazzaley and Nobre, 2012, for a review). Neural evidence further shows that the same neural systems, including the superior parietal lobules, are involved in top-down attentional control in working memory and attentional modulation during perception (Ruff et al., 2007). Accordingly, presentation of the retro-cue in partial report may have shifted selective attention on the target letter representation in memory, thus resulting in similar performance whatever the position of the target within the string. Since attention modulation by retro-cues is task-determined, all target letters had the same probability to be accurately reported whatever the participant's language. More investigation is needed to better understand the potential role of task-dependent VA strategies in string processing.

### VA span and reading speed relationship

Lastly, we measured text-reading speed and explored whether reading performance correlated with VA span abilities in all three languages. Direct cross-language comparison of text reading speed is rather risky, as the Arabic text was not strictly matched to the French and Spanish texts. Nevertheless, as typically reported (Azzam, 1993; Abu-Rabia, 2001; Abu Ahmad et al., 2014), the skilled adult readers of Arabic were found to read more slowly than the French or Spanish participants. Slower reading in Arabic cannot reflect cross-language differences in transparency. Such an account would have predicted quite different reading speed in French and Spanish. Some difference does exist but goes the other way round, Spanish readers being slightly more fluent than French readers.

Some insights from other languages (Rao et al., 2011) suggest that letter visual complexity affects reading speed, which could contribute to slower reading in Arabic. The current findings do not support this hypothesis since single letter identification did not correlate with text reading speed performance. It is also possible that greater reliance on context in skilled Arabic readers (Abu-Rabia, 1997) requires meaning computation to be completed for successful word identification. Such additional processing might affect reading speed, contrary to European languages in which skilled readers rely on orthographic processing with no or minimal influence of context (Nicholson, 1991). The absence of whole word reading strategy (Eviatar and Ibrahim, 2014) and additional time needed to process internal morphemes (Boudelaa, 2014) within words are further factors that could affect reading fluency in Arabic.

Last, we examined the relationship between text reading speed and VA span abilities in all three languages. A significant relationship was found for French but not for Spanish or Arabic, which could reflect differences in orthographic transparency. These findings differ from those previously reported in children. Indeed, VA span skills are tightly related to reading speed in children from both shallow and deep orthographies, including Spanish (Lallier et al., 2014) and French (Lobier et al., 2013). Similar evidence is lacking for Arabic children. Overall, the available data suggests that VA span abilities relate to text reading performance in French and Spanish (and potentially Arabic) children but that adult French readers alone show a significant relationship. Longitudinal or cross-sectional studies are needed to confirm a potential modulation of VA span influence on reading speed performance depending on the participant's age and language transparency.

To conclude, the current findings suggest that VA span may differ in the European and Semitic languages. Evidence for a smaller VA span in Arabic questions its potential extension to other Semitic languages, like Hebrew, and the origin of differences in the size of VA span in these two language families. Evidence for a left-right asymmetry potentially related to factors associated with reading direction when the task involves the identification of all of the letters within string but the absence of asymmetry when attention is manipulated by a retro-cue provides new insights for better understanding the mechanisms involved in asymmetry effects. Last, the relationship between VA span and reading may be differently modulated by age depending on the language characteristics. A deeper exploration of this latter issue is critical to better understand how the reading system develops in different linguistic contexts.

## Author contribution

Conceived and designed the experiment: SV, FA; Performed the experiments: FA, AA; Analyzed the data: TP, FA; Interpretation of the data: FA, SV, MC, TP, ML, AA; Contribution to the redaction: SV, FA, TP, ML, MC.

## Funding

FA was supported by a fellowship from the Iraqi Ministry and Campus-France, TP by a Ph.D grant from the “Fondation de France” and AA by a Basque Government PhD grant. The study was supported by the ANR (ANR-12-BSH2-0013-01) grants to SV, the European Research Council (ERC advanced grant, Proposal No. 295362, BILITERACY awarded to MC) and the Spanish Government (Plan Nacional-PSI2012-32128 awarded to ML, and Plan Nacional-PSI2012-31448 awarded to MC).

### Conflict of interest statement

The authors declare that the research was conducted in the absence of any commercial or financial relationships that could be construed as a potential conflict of interest.

## Footnotes

^1^The 10 Arabic letters include the letter “Ya”' that is classified as a consonant in the Arabic alphabet and acts as a consonant when processed in isolation. However, Ya' often acts as a vowel in context (Saiegh-Haddad and Joshi, 2014).

^2^Significant correlations were also found in the current study for French (*r* = 0.36, *p* = 0.021), Spanish (*r* = 49, *p* = 0.0008) and Arabic (*r* = 0.37, *p* = 0.014).
