# Supplementary material for: Cross-Language Modulation of Visual Attention Span: An Arabic-French-Spanish Comparison in Skilled Adult Readers
Source: Front Psychol. 2016 Mar 7;7:307. doi: 10.3389/fpsyg.2016.00307 (PMC4779959; doi:10.3389/fpsyg.2016.00307)
Supplement: Supplementary file 1 [file DataSheet1.pdf]

- **The French Text**

A Moscou, le « refuge » classé mais délaissé de la Maison rouge

C'est une grande bâtisse de briques rouges, cachée derrière les immenses arbres d'un jardin anarchique. Aujourd'hui, ses habitants ne puisent plus l'eau au puits et ils ont l'électricité, des immeubles de banlieue ont poussé tout autour et le métro n'est pas loin. Ce n'est plus une maison à la campagne et pourtant le temps s'est arrêté là, entre ces murs lézardés qui ont abrités des générations d'artistes et abritent toujours leurs descendants.

La maison rouge pourrait être un musée, dans cette Russie qui les aime tant, et où tout est prétexte à en créer. Mais la volonté de l'un de ses deux fondateurs a été respectée : elle est restée maison d'habitation. Au-dessus des trois ateliers du rez-de-chaussée vivent plusieurs familles, générations mêlées, comme il est toujours d'usage dans le pays. De Dimitri, 74 ans, à Dimitri, son arrière-petit-fils, 3 ans, dix-huit personnes l'habitent.

Dix-huit personnes et un siècle d'histoire, dont les murs sont chargés. A la fin des années 1930, une grande exposition agricole est organisée à Moscou. Certains des artistes qui mettent la main à la pâte reçoivent pour paiement…un terrain. Sur le leur, à une quinzaine de kilomètres du centre de la capitale, Vladimir Favorski (1886-1964) et Ivan Efimov (1878-1959) construisent, en 1939, ce qui restera pendant des décennies une des rares propriétés privées de Moscou. Tous deux sont issus de familles de la vieille noblesse. Le premier est connu surtout comme illustrateur de livres, le second comme sculpteur animalier. Mais ils touchent à tout.

Au début du siècle, Ivan Efimov et sa femme ont arpenté la Russie avec un théâtre de marionnettes et un théâtre d'ombres ambulants. Ils peignent, sculptent, gravent, dans cette Maison rouge qui leur a permis de rassembler leur famille. Vladimir Favorski vivait jusque-là dans un appartement communautaire dans le centre, tandis que femme et enfants, chassés par la famine, avaient quitté la ville. La demeure est vaste, elle sert de refuge aux amis, qui investissent jusqu'au grenier. Dans les années 1960, Dimitri Jilinski, aujourd'hui l'un des plus célèbres peintres russes, y verra naître ses deux enfants.

Des amis musiciens viennent répéter. Tout est prétexte à spectacles, bals masqués, soirées théâtrales dans ce lieu qu'à l'époque on atteint, l'hiver, après une longue marche dans la neige, et qui ébahit le visiteur venu bien souvent d'un appartement communautaire. Aujourd'hui, dans les ateliers, dans les pièces d'habitation, les murs sont couverts des œuvres de Favorski, de Efimov, de leurs femmes, de leurs enfants, de leurs gendres, de leurs petits-enfants et arrière-petits-enfants, et même des aïeux des pièces « rapportées », issues elles aussi de l'intelligentsia du XIXe siècle. Des tableaux qui appartenaient à la princesse Cheremetiéva – une grande famille de l'époque tsariste dont certains descendants vivent à la Maison rouge – aux créations des plus jeunes habitants, la qualité est inégale. Mais l'ensemble témoigne de la vitalité des milieux artistiques dans le Moscou du XXe siècle.

« Il ne s'agit pas seulement d'un capital plastique, mais aussi d'un capital moral », affirme Valentina Alexeievna, qui vient d'organiser une exposition sur la Maison rouge dans la ville de Podolsk, à une vingtaine de kilomètres de Moscou. « C'est un endroit unique, dit-elle parce qu'il puise à des racines très profondes, les aïeux faisaient partie de la vieille intelligentsia éclairée, la belle-mère de Favorski avait créé le premier jardin d'enfants qui ait existé à Saint-Petersbourg, son mari, médecin, soignait les pauvres et recueillait les orphelins ».

Deux patriarches ont succédé à Vladimir Favorski et Ivan Efimov : les époux de leurs petites-filles, Dimitri Chakhnovskoï et Illarion Golitsine. Le premier, sculpteur, aujourd'hui âgé de 74 ans, gagnait sa vie en travaillant notamment pour des théâtres. Il est l'auteur de l'immense horloge apposée sur la façade du grand théâtre de Marionnettes de Moscou, et certaines de ses œuvres figurent dans les plus grands musées de Russie. Ce qui ne l'empêche pas de vivre dans la plus grande simplicité, et de désespérer de trouver les fonds qui permettraient de rénover la Maison rouge, classée au patrimoine mais délaissée. « On a grandi dans un tel milieu dit-il qu'on ne peut pas voir l'art comme un commerce ».

- ## The Spanish Text

Los ciclones se van hacia los polos

Muchas regiones del planeta, especialmente en el Pacífico y en el Índico, que solían estar a salvo de los devastadores efectos de los ciclones tropicales, tienen cada vez más riesgo de ser asoladas periódicamente por estas gigantescas tormentas especialmente intensas. El tifón Haiyan (2013), o los huracanes Katrina (2005) y Sandy (2012) son dramáticos ejemplos. La actividad de los ciclones tropicales, desde hace 30 años, se está desplazando desde la banda tropical hacia los polos al buen ritmo de algo más de 50 kilómetros por década (53 y 62 kilómetros en los hemisferios Norte y Sur respectivamente). A la vez que la fase de máxima intensidad de las tormentas migra hacia latitudes altas y amenaza a las zonas costeras allí, el riesgo puede ser menor en las regiones tropicales que tradicionalmente soportan tifones y huracanes. Pero este potencial alivio puede a la vez convertirse en un problema ya que estas tormentas de vientos huracanados y generadoras de inundaciones son clave para alimentar las reservas de agua en esas regiones.

Pese a que hay muchas incógnitas aún acerca de los mecanismos y la futura evolución de esta migración de los ciclones, los científicos constatan que el fenómeno coincide con la conocida expansión de los trópicos hacia los polos, fenómeno que se ha relacionado, al menos en parte, con el cambio climático inducido por la actividad humana. “Ahora que vemos esta clara tendencia, es crucial comprender sus causas de forma que podamos anticipar qué va a ocurrir en los próximos años y décadas”, señala Gabriel Vecchi, científico de la Agencia Nacional de Océano y Atmósfera (NOAA) estadounidense.

James Kossin (NOAA), Vecchi y Kerry A. Emmanuel (científico del Instituto de Tecnología de Massachusetts), han analizado la tendencia de los ciclones tropicales localizando dónde alcanza cada uno su máxima intensidad. Así han descubierto el proceso de migración hacia latitudes cada vez más altas. Y este desplazamiento, afirman en su artículo en la revista *Nature*, “puede relacionarse con la expansión tropical que se considera tiene una contribución antropogénica”.

Las tormentas tropicales intensas o ciclones (denominados tifones en la región del Pacífico occidental y huracanes en el Atlántico) son fenómenos que se originan en condiciones de bajas presiones, altas temperaturas del agua oceánica, máxima humedad y no mucha diferencia entre las velocidades del viento en la baja y alta troposfera. Los requisitos para su formación se dan, precisamente en los trópicos y se desarrollan y desplazan alimentados por el agua del mar, por lo que se desvanecen al poco de llegar a las regiones costeras, donde producen, al entrar, daños devastadores sobre todo si la población no está preparada y protegida. Estas condiciones imprescindibles impiden que el desplazamiento hacia los polos observado continúe indefinidamente, señala Hamish Ramsay, experto de la Universidad Monash (Australia), al comentar el trabajo de Kossin y sus colegas. Sencillamente llega un momento en que el régimen de vientos y la esencial temperatura alta del agua (unos 26 grados) impiden la supervivencia del ciclón tropical, a no ser que se den “cambios no plausibles de las restricciones físicas fundamentales en la circulación atmosférica, como la tasa de rotación de la Tierra”, ironiza Ramsay.

Pese a que los ciclones tropicales se conocen bien y se observan y vigilan perfectamente, lo que facilita la alerta para que las zonas de riesgo puedan prepararse, en la investigación del cambio climático vienen siendo una pesadilla, hasta el punto de que en el último informe del Panel Intergubernamental sobre Cambio Climático (IPCC) de Naciones Unidas se clasifica con “un nivel de confianza bajo” la predicción para el futuro de una mayor intensidad de la actividad de estos fenómenos. Un problema es que su frecuencia es relativamente baja como para poder discernir claramente una tendencia en las décadas recientes, en las que se tienen datos precisos sobre los ciclones.

“Hace falta investigar más y a más largo plazo para poder determinar si el desplazamiento hacia los polos de la máxima intensidad de los ciclones puede ser relacionada con la actividad humana”, advierten ahora muy prudentemente los investigadores de la NOAA. Y el fenómeno no es uniforme, según los resultados de Kossin, Emanuel y Vecchi, con la máxima migración observada en los últimos 30 años en el Pacífico Norte y Sur, así como en el Índico, mientras que no se observa esta tendencia en el Atlántico.

- **The Arabic Text**

العلامة طه باقر (قارئ الطين)

تتفاخر الأمم والشعوب بمبدعيها وعلمائها ومفكريها، وتعمل من اجل ان يبقوا في ذاكرة التاريخ، ولكي يكونوا منارة تَسترشد بها الاجيال, ويكونوا معينا لهم في دروب المعرفة. و في تاريخ وادي الرافدين هناك شخصيات خالدة نعتز بها نَقشَت أسماءها في طين هذا البلد، وامتزجت مع مائهِ وترابهِ بنصوصٍ سحرية مقدسة, فدخلت القلوب وعشقتها الأفئدة, ومن هذه الشخصيات الخالدة الأستاذ طه باقر الملقب(قارئ الطين) الذي أصبحَ محطَ فخرٍ لتلك الحضارة التي أنجبته, وقدّرت عليه مهمة اكتشافها وتنقيبها وترجمة أساطيرها , فمنذ ان كان طفلاً صغيراً سارَ على أرضِ بابل، و استلهمَ منها عبقَ الماضي العظيم, ونفحات ملوكها وآلهتها, فلم تَغب عن ذاكرتهِ بوابةُ عشتار.

شَهدت الفترة الاولى من تكّون الدولة العراقية ظاهرة فريدة في مجال التأسيس المعرفي ،وبرزت فيها نخبة من المؤسسين تركوا بصماتهم في كل المجالات التي عملوا فيها، ومن هذهِ النخبة المؤرخ الآثاري العلامة طه باقر.

ولدَ الأستاذ طهَ باقر في محافظة بابل عام 1912 في مدينه الحلة ,وعاش في بيئة علمية، إذ إن والده وعمّه كانا من المعروفين باهتماماتهم الدينية، لذلكَ حَرصا على أن يلتحق بالمدارس الدينية، ويدرس على الطريقة الكلاسيكية اللغة والنحو والصرف والبلاغة وشيئا من الحساب.

أكمل دراستهُ الابتدائية والمتوسطة في نفس المدينة وكان من الطلبة المتفوقين دراسيا، واكملَ الثانوية في بغداد على نفقة وزارة المعارف في الثانوية المركزية (الفرع العلمي)عام، (1931-1933) بتفوق وتَميز وكان واحداً من الاربعة الاوائل في ثانويات العراق، واختير ضمن طلاب البعثة التي كانت ترسلها وزارة المعارف لإكمال الدراسة خارج العراق، وكانت بعثته لدراسته علم الآثار. وبعد أن حصل على شهادتي البكالوريوس والماجستير عادَ إلى العراق عام 1938.

للأستاذ طه باقر إسهاماتٌ فاعلة في تهيئة الكُتب المدرسية المقررة للتدريس في المدارس المتوسطة، وهو موضع تقدير واحترام زملائهِ وتلاميذهِ والذين اهتموا بتراثه الفكري. فلقد كُتب عنّه كثيراً. كما أٌلِف كتابٌ عن سيرتهِ ومنجزاتهِ، وأشيد بقدراته العقلية وميزاتهِ العلمية وذهنيتهِ المتفتحة فضلاً عن طيبته وجديته وحّبه لعمل الخير. إن تلك ألإسهامات كثيرة جداً ويصعبُ إحصاؤها لغزارة الإنتاج الفكري الذي كان يسهم به المرحوم الأستاذ طه باقر. وكانت كل مقالة عبارة عن بحثٍ علمي مُتكامل وإسهامة حقيقية في مجال التأريخ والتراث والآثار.

يعد طه باقر من ألمع العاملين في مجال ترميم الذاكرة العراقية وصلتها بتاريخها الحيوي المتحرك الحي المنتج، وعمل في مجال التاريخ القديم وعلى الأخص تاريخ العراق, واهتم بإعادة صورة الجماعة العراقية المنتجة والجدية في تفاعلها مع بيئتها الطبيعية والاجتماعية. لقد كانت الدولة العراقية الحديثة التي عاش بداياتها وفتوتها العلامة طه باقر مدعاة للنظر في تاريخ الإنسان العراقي وكيف أبدع المع حضارات العالم القديم. وكانت وفاته في عام1984.
